# The role of a tantalum interlayer in enhancing the properties of Fe_3_O_4_ thin films

**DOI:** 10.3762/bjnano.15.101

**Published:** 2024-10-14

**Authors:** Hai Dang Ngo, Vo Doan Thanh Truong, Van Qui Le, Hoai Phuong Pham, Thi Kim Hang Pham

**Affiliations:** 1 Faculty of Applied Sciences, Ho Chi Minh University of Technology and Education, 720700 Ho Chi Minh City, Vietnam; 2 Department of Materials Science and Engineering, National Tsing Hua University, Hsinchu 300093, Taiwan; 3 NTT Hi-Tech Institute, Nguyen Tat Thanh University, 298-300A Nguyen Tat Thanh Street, Ward 13, District 4, Ho Chi Minh City 700000, Vietnam

**Keywords:** buffer layer, Fe_3_O_4_, magnetite, RF magnetron sputtering, spintronic

## Abstract

High spin polarization and low resistivity of Fe_3_O_4_ at room temperature have been an appealing topic in spintronics with various promising applications. High-quality Fe_3_O_4_ thin films are a must to achieve the goals. In this report, Fe_3_O_4_ films on different substrates (SiO_2_/Si(100), MgO(100), and MgO/Ta/SiO_2_/Si(100)) were fabricated at room temperature with radio-frequency (RF) sputtering and annealed at 450 °C for 2 h. The morphological, structural, and magnetic properties of the deposited samples were characterized with atomic force microscopy, X-ray diffractometry, and vibrating sample magnetometry. The polycrystalline Fe_3_O_4_ film grown on MgO/Ta/SiO_2_/Si(100) presented very interesting morphology and structure characteristics. More importantly, changes in grain size and structure due to the effect of the MgO/Ta buffering layers have a strong impact on saturation magnetization and coercivity of Fe_3_O_4_ thin films compared to cases of no or just a single buffering layer.

## Introduction

Magnetite, also known as Fe_3_O_4_, has been extensively researched as one of the most common half-metallic materials in the field of spintronics for a considerable period of time. Magnetoelectronic devices are possible because of the material’s high Curie temperature of 860 K [1], as well as its high spin polarization with only one spin at the Fermi level, even at room temperature [2–6]. Fe_3_O_4_ thin films are an issue of interest and have extensive applications in Li-ion batteries, spin Seebeck devices, supercapacitors, spin Hall magnetoresistance, and the study of analog resistive switching of Fe_3_O_4_-based cross-cell memristive devices [7–10].

Fe_3_O_4_ thin films can be grown by many processes, including molecular beam epitaxy, which is employed for depositing single crystal films, and pulsed laser deposition, which is utilized to achieve epitaxial films [11–13]. The RF magnetron sputtering technique is extensively utilized because of its cost-effectiveness, simplicity, effectiveness, and capacity to produce Fe_3_O_4_ films with remarkable uniformity. The qualities of the films can be modified by manipulating parameters throughout the growth process [14–15]. The impact of substrate temperature, annealing temperature, gas flow rate, and thickness on enhancing the characteristics of Fe_3_O_4_ thin films has been examined [15–18]. The substrates play a crucial role in directing the growth and enhancing the quality of the crystal, resulting in significant changes in the film’s characteristics [19–20].

Roy et al. conducted a study on polycrystalline Fe_3_O_4_ films on Si and SiO_2_/Si substrates. Their findings revealed that the value of the Gilbert damping parameter is significantly higher in Fe_3_O_4_/Si films compared to Fe_3_O_4_/SiO_2_/Si films [21]. Hong and coworkers deposited Fe_3_O_4_ films on a MgO substrate, which exhibited a change in the direction of Fe_3_O_4_ crystal formation. The directions (222), (400), and (440) of the Fe_3_O_4_ peak matched, respectively, the (111), (100), and (110) orientations of the MgO substrate [22]. In addition, Zhang et al. successfully applied a layer of Fe_3_O_4_(001) on a MgO(001) substrate. The resulting material exhibited saturation magnetization and magnetic moment values of 407 ± 5 emu/cm^3^ (3.26 ± 0.04 μ_B_/(f.u.)) and 3.31 ± 0.15 μ_B_/(f.u.), respectively [23].

This paper addresses the deposition of Fe_3_O_4_ thin films on three different types of substrates, namely an amorphous SiO_2_/Si(100) substrate, a single crystal MgO(100) substrate, and a buffer layer consisting of MgO/Ta/SiO_2_/Si(100). The properties of Fe_3_O_4_ thin films were analyzed using atomic force microscopy (AFM), X-ray diffractometry (XRD), and vibrating sample magnetometry (VSM). It is interesting to note that the saturation magnetization of the Fe_3_O_4_ films was significantly improved (278.9 emu/cm^3^) when utilizing a Ta interlayer located between MgO and SiO_2_, compared to films on SiO_2_ (136.3 emu/cm^3^) and MgO(100) (126.3 emu/cm^3^) substrates. This indicates the potential to facilitate the development of novel magnetic and spintronic architectures.

## Results and Discussion

**Figure 1:**
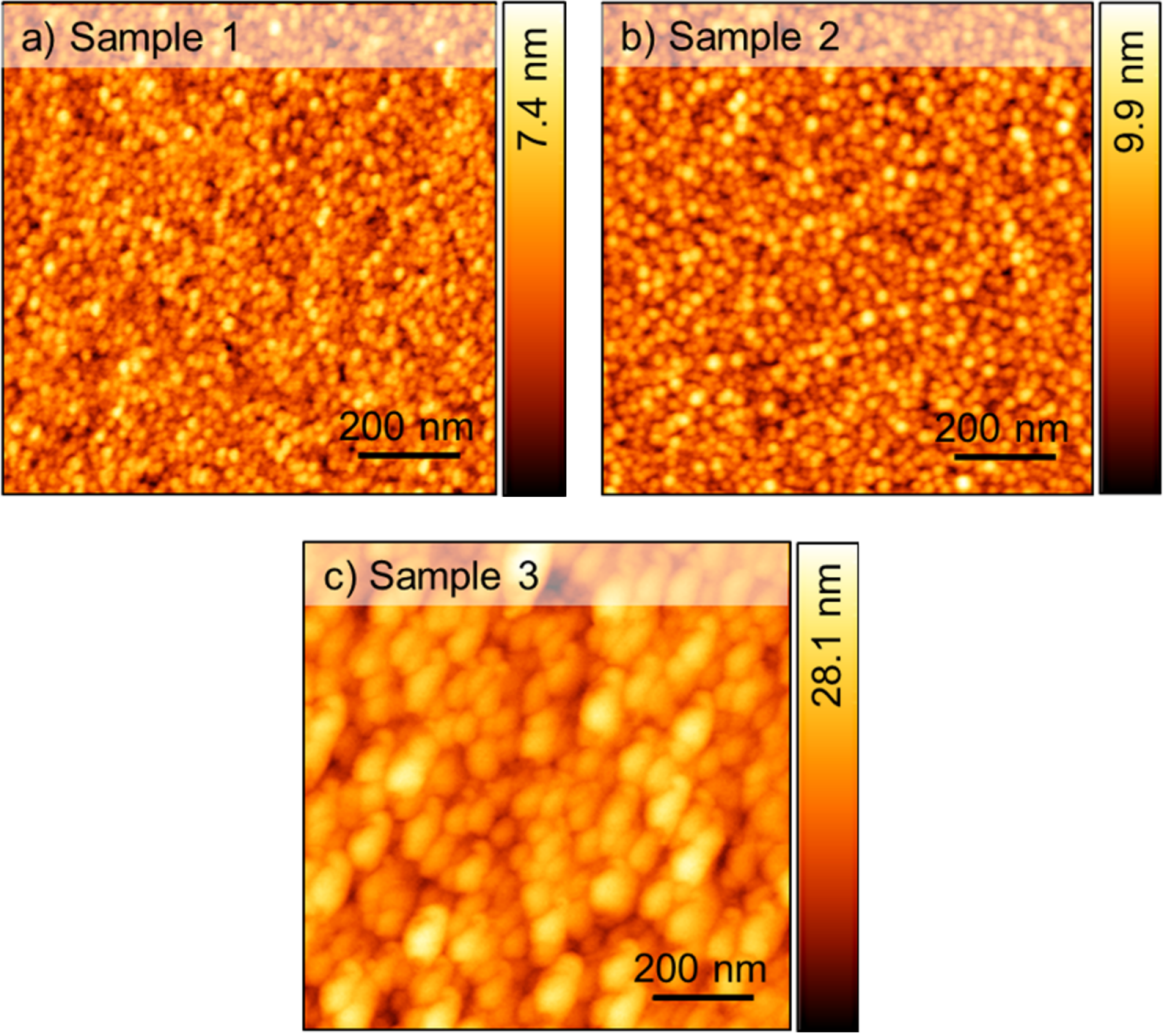
AFM images (1 × 1 µm^2^) of Fe_3_O_4_ thin films on different substrates. (a) SiO_2_, (b) MgO(100), and (c) MgO/Ta/SiO_2_.

AFM and line-cut method were used to examine the surface morphology and grain sizes of the Fe_3_O_4_ films that were formed on SiO_2_/Si(100), MgO(100), and MgO/Ta/SiO_2_/Si(100) multilayer substrates (referred to as samples 1, 2, and 3, recpectively). Topography images, with dimensions of 1 × 1 μm^2^, are shown in Figure 1. They show spherical particles with rather consistent grain sizes. In particular, samples 1 and 2 present grain size values of 7.6 ± 0.5 nm and 9.9 ± 0.6 nm, respectively. Sample 3, grown on the MgO/Ta/SiO_2_ multilayer structure, reveals the largest value of 31.4 ± 1.4 nm. In addition, the Fe_3_O_4_ samples present quite different root-mean-square (RMS) roughness values of 0.94 ± 0.09 nm, 1.29 ± 0.14 nm, and 3.58 ± 0.58 nm for samples 1, 2 and 3, respectively. Sample 3 with the highest value has the roughest surface among the three. These results indicate that the substrate type does have an effect on grain size and roughness of Fe_3_O_4_ thin films. Tantalum in the multilayer structure prevents the diffusion of oxygen atoms from SiO_2_ into MgO leading to enhanced stability of MgO [24–25]. Besides, there was nearly no oxygen diffusion from the Fe_3_O_4_ film into the MgO layer, resulting in higher crystallinity and improved grain size as seen in the XRD patterns. Surface properties obtained from Figure 1 are summarized in Table 1.

**Table 1:** Surface properties obtained from the AFM scans of Fe_3_O_4_ samples.

	RMS roughness (nm)	Grain size (nm)	Peak-to-valley height (nm)

sample 1	0.94 ± 0.09	7.6 ± 0.5	4.9 ± 0.6
sample 2	1.29 ± 0.14	9.9 ± 0.6	6.6 ± 0.9
sample 3	3.58 ± 0.58	31.4 ± 1.4	16.4 ± 2.9

**Figure 2:**
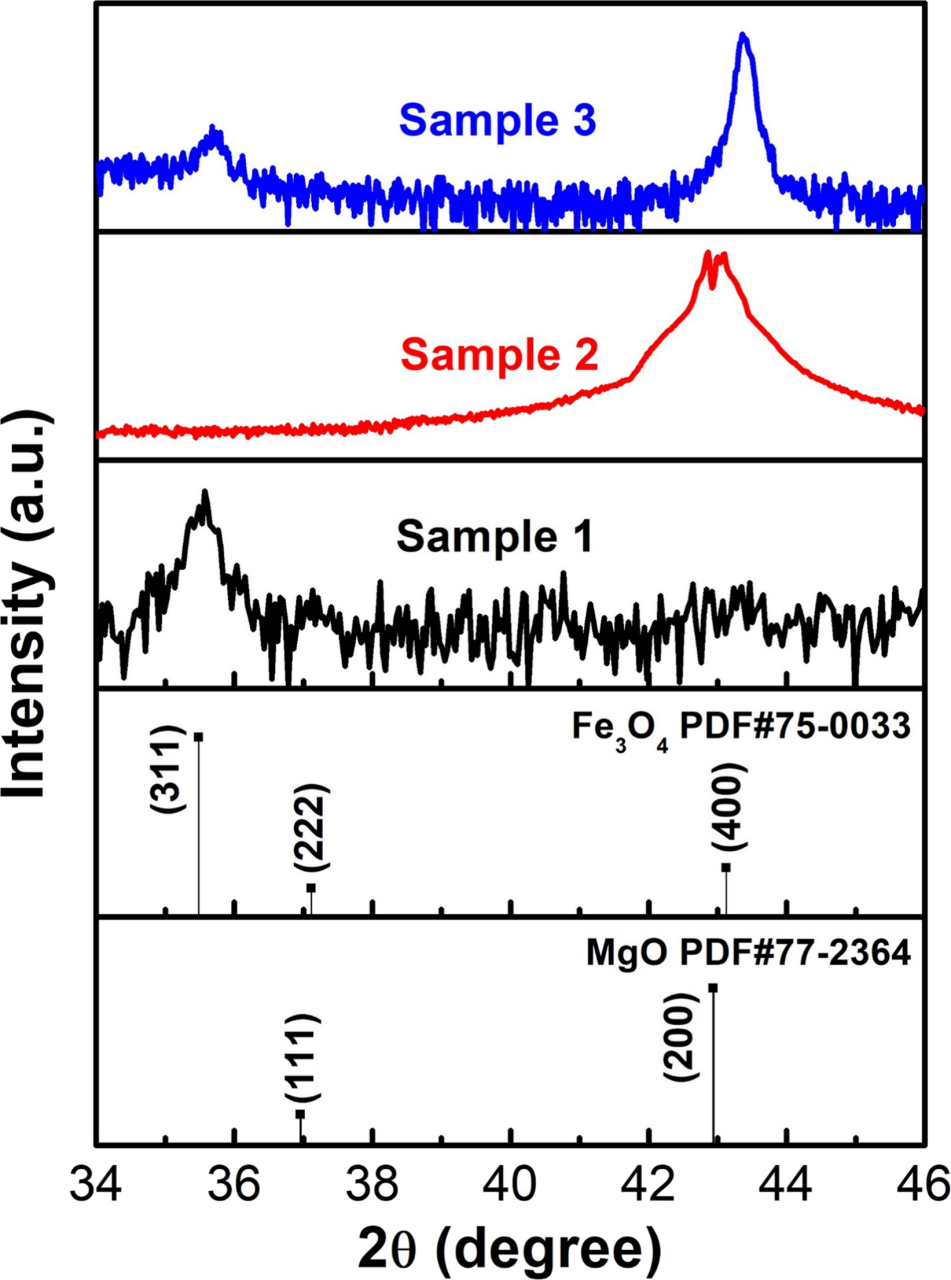
XRD spectra of sample 1 (black), sample 2 (red), and sample 3 (blue) on SiO_2_, MgO(100) and MgO/Ta/SiO_2_, respectively.

The crystal structures of the Fe_3_O_4_ samples on different substrates were investigated with XRD measurements, and the corresponding diffraction patterns are depicted in Figure 2. The Fe_3_O_4_ sample grown on the SiO_2_/Si(100) substrate exhibits a single Fe_3_O_4_(311) peak located at 35.5° (black line), while the one deposited on MgO(100) exhibits the Fe_3_O_4_(400) peak at 43.07° (red line). This indicates the epitaxial growth of the Fe_3_O_4_ thin film on MgO(100). To our surprise, the Fe_3_O_4_ thin film deposited on the multilayer structure shows the two peaks Fe_3_O_4_(311) and Fe_3_O_4_(400) at 35.68° and 43.36°, respectively (blue line). This implies that the tantalum interlayer has an effect on the crystallization of the Fe_3_O_4_ film.

XRD patterns provide further information about the structural properties of a material, such as lattice constant (*a*), dislocation density (δ), and microstrain (ε). Bragg’s law was used to calculate the *d*-spacing of the Fe_3_O_4_(311) and Fe_3_O_4_(400) peaks [26–27]:


[1] nλ=2dsinθ,

where *n* is the order of diffraction (*n* = 1) and λ is the X-ray wavelength (Cu Kα, λ = 1.5406 Å). The lattice constant *a* of the three Fe_3_O_4_ samples was determined by [27–28]:


[2] $$\frac{1}{d_{\left(hkl\right)}}=\frac{\sqrt{h^{2}+k^{2}+l^{2}}}{a}.$$

The microstrain in these samples can be calculated from the lattice constant *a* above by using the following relation [27–28]:


[3] $$\varepsilon =\frac{a-a_{0}}{a_{0}}\times 100,$$

where *a*_0_ is the lattice parameter of bulk Fe_3_O_4_ (*a*_0_ = 8.397 Å [29]). Microstrain is a crucial factor that helps to analyze the existence of strain and deformation in thin films [30–31].

The *d*-spacing values of the Fe_3_O_4_(311) and Fe_3_O_4_(400) peaks of sample 3 are 2.514 and 2.085 Å, respectively, which are smaller than those on SiO_2_ (2.527 Å) and MgO(100) (2.099 Å) substrates. These low *d*-spacing values can be caused by the microstrain in all Fe_3_O_4_ samples [27–28]. The Fe_3_O_4_ film grown on the multilayer structure is under a higher compressive strain of −0.70% and −0.67%, corresponding to the Fe_3_O_4_(311) and Fe_3_O_4_(004) peaks, respectively, than samples 1 and 2 with values of −0.19% and −0.01%, respectively. Sample 3 exhibits a decrease in *d*-spacing for both the (311) and (400) peaks, in comparison to sample 1 and sample 2. The presence of compressive stress in the crystallites of the Fe_3_O_4_ thin films causes a shift in the peak observed in sample 3 [32]. Our results reveal that the growth orientation of the Fe_3_O_4_ thin film depends on the lattice mismatch between the Fe_3_O_4_ thin film and the substrate or buffer layer. When the Fe_3_O_4_ thin film is deposited on the amorphous SiO_2_ substrate, the lattice mismatch between the amorphous substrate and the crystalline film is large. In this case, the growth orientation of Fe_3_O_4_ thin film is determined by the direction having the least internal energy, which is [111]. The energetically favored [111] direction also has the highest probability of occupying random dangling bonds from the amorphous substrate surface because it has the highest areal density [13,33]. In contrast, the small lattice mismatch between Fe_3_O_4_ thin film and MgO(100) substrate (≈0.3% [29]) results in the growth orientation controlled by the substrate and leads to the appearance of the [100] direction in Fe_3_O_4_/MgO. In addition, the Fe_3_O_4_(400) and MgO(200) peaks are close because of the similarity in crystalline structure (cubic) and lattice constant (*a*_Fe3O4_ = 8.397 Å, *a*_MgO_ = 4.212 Å [29]). The growth orientation of the Fe_3_O_4_ thin film in sample 3 is also affected by the internal energy of the [111] direction in addition to effects from the buffer layer. This explains the highest microstrain value in sample 3. The difference in lattice constants between MgO and Ta (cubic, *a*_Ta_ = 3.3058 Å [34]) puts the MgO buffer layer under a higher strain and creates a larger lattice mismatch between the Fe_3_O_4_ thin film and the MgO layer compared to the Fe_3_O_4_ thin film and MgO substrate.

In addition, the dislocation density was calculated by the following relation [31]:


[4] $$\delta =\frac{1}{D^{2}},$$

where *D* is the crystallite size, which can be found by using the Scherrer equation. The dislocation density of sample 1 is the highest, 6.6 × 10^−4^ nm^−2^, resulting from oxygen atoms Fe_3_O_4_ occupying random dangling bonds of the SiO_2_ surface [13,33]. In contrast, Fe_3_O_4_ thin films deposited on MgO have a low dislocation density of 0.8 × 10^−4^ nm^−2^ for MgO substrate and 1.9 × 10^−4^ nm^−2^ and 0.9 × 10^−4^ nm^−2^ for MgO with Ta buffer layer. The microstructural properties are summarized in Table 2.

**Table 2:** Structural parameters of Fe_3_O_4_ thin films on various substrates.

	Fe_3_O_4_ peaks	*d*-spacing (Å)	*a* (Å)	ε (%)	δ (10^−4^·nm^−2^)

sample 1	(311)	2.527	8.381	−0.19	6.6
sample 2	(400)	2.099	8.396	−0.01	0.8
sample 3	(311)	2.514	8.338	−0.70	1.9
	(400)	2.085	8.340	−0.67	0.9

**Figure 3:**
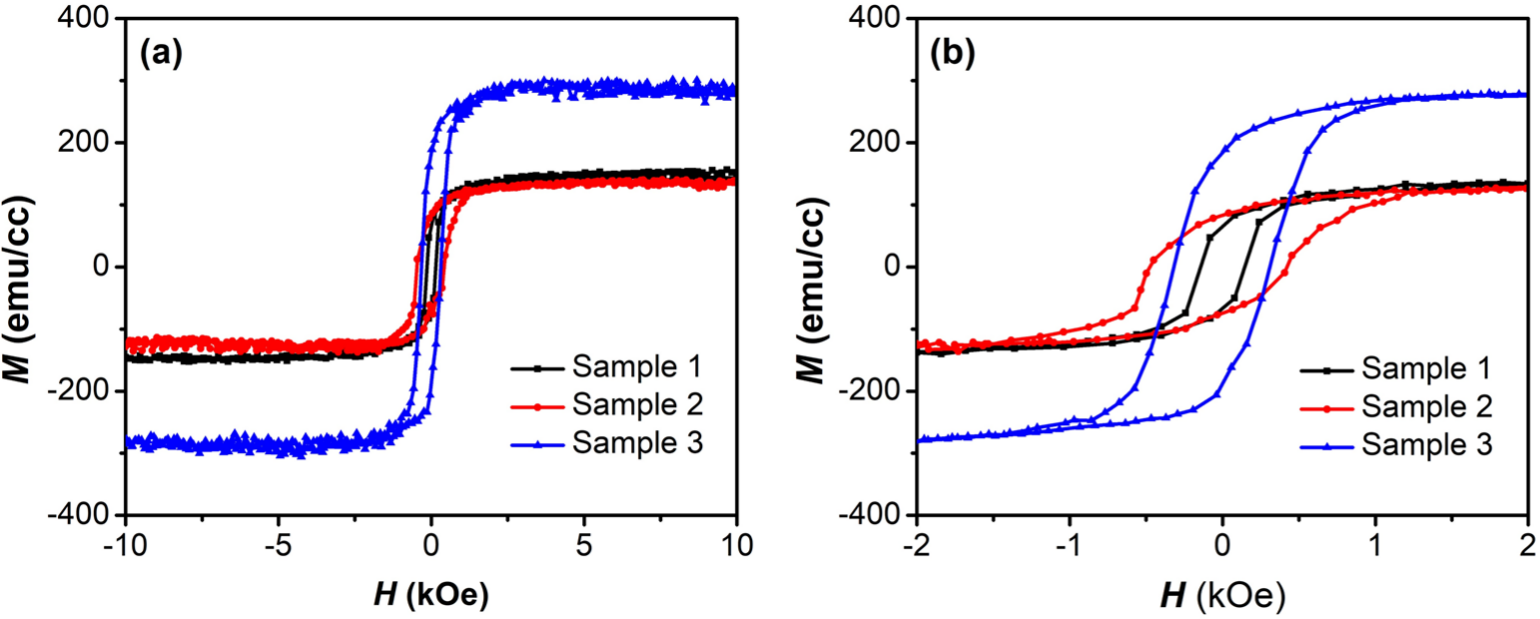
Magnetization of Fe_3_O_4_ samples in an external magnetic field in a range of (a) −10 kOe to 10 kOe and (b) −2 kOe to 2 kOe.

To characterize the effect of microstructure and morphology on the magnetic properties of Fe_3_O_4_ thin films, VSM measurements were conducted in an external field from −10 kOe to 10 kOe at room temperature. Figure 3 depicts the hysteresis curves (*M*–*H*) of samples 1, 2, and 3. The magnetization of the Fe_3_O_4_ thin film grown on the multilayer structure is significantly larger than the those of the others, as shown in Figure 3a. Figure 3b shows a magnification of the *M*–*H* loops from −2 kOe to 2 kOe to show more details. The remanent magnetization (*M*_r_) of sample 3 is the largest, 180.9 emu/cm^3^, while the *M*_r_ values of samples 1 and 2 are 66.8 and 84.3 emu/cm^3^, respectively. All Fe_3_O_4_ thin films exhibit saturation at 2 kOe, which is smaller than the values given in other reports [35–36]. The Fe_3_O_4_/MgO/Ta/SiO_2_ sample has a saturation magnetization (*M*_s_) of 278.9 emu/cm^3^, which is dramatically higher than that of the Fe_3_O_4_ thin films on SiO_2_ (136.3 emu/cm^3^) and on MgO (126.3 emu/cm^3^). The coercivity (*H*_c_) of sample 1 is 142.2 Oe, while the *H*_c_ values of samples 2 and 3 are 421.2 and 310.1 Oe, respectively. The remanence ratio (*M*_r_/*M*_s_) indicates the amplitude of exchange coupling in Fe_3_O_4_ thin films. The results reveal that the remanence ratios of Fe_3_O_4_ thin films grown on MgO are larger than that on SiO_2_. The stronger the exchange coupling, the larger the remanence ratio [37]. The magnetic parameters of the Fe_3_O_4_ samples are summarized in Table 3.

**Table 3:** Magnetic parameters of samples 1, 2, and 3.

	*M*_r_ (emu/cm^3^)	*M*_s_ (emu/cm^3^)	*M*_r_/*M*_s_	*H*_c_ (Oe)

sample 1	66.8	136.3	0.49	142.2
sample 2	84.3	126.3	0.67	421.2
sample 3	180.9	278.9	0.65	310.1

Morphology, microstructure, and anisotropy mechanisms significantly impact the magnetic properties of ferrite materials [38]. It is known that antiphase boundaries (APBs) and grain size in Fe_3_O_4_ thin films, which are strongly influenced by substrate or buffer layer, can affect *M*_s_ [39–40]. In reports [41–42], APBs in Fe_3_O_4_ thin films lead to the reduction of saturation magnetization compared to bulk Fe_3_O_4_ (510 emu/cm^3^) [29]. Therefore, sample 3 has the highest *M*_s_ because of the smallest number of APBs among all samples. Using a double buffer layer of MgO/Ta to lower the crystallization temperature can help to reduce the number of APBs in Fe_3_O_4_ thin films [43]. Besides, the increased grain size in sample 3 also results in an increased *M*_s_ as described in [44]. The change in *H*_c_ also depends on two factors, that is, APBs and grain size [45]. Thanks to the prevention of oxygen diffusion of the Ta buffer layer, sample 3 has the largest grain size (Table 1). These large intergranular regions can enhance the number of magnetic moments, making it harder for them to rotate when an external field is applied. Although the grain size in Fe_3_O_4_ thin films on the double buffer layer is nearly 3.5 times larger than that in sample 2, the number of APBs in sample 3 is the smallest, resulting in the reduction in *H*_c_ of sample 3 compared to the *H*_c_ value of sample 2.

## Conclusion

Fe_3_O_4_ films were prepared on different substrates of SiO_2_/Si(100), MgO(100), and MgO/Ta/SiO_2_/Si(100) at room temperature using RF sputtering. Our finding highlights the role of the Ta buffer layer in the multilayered structure. Ta helps to decrease the crystallization temperature of the Fe_3_O_4_ film and prevents the diffusion of oxygen atoms from SiO_2_ to MgO, resulting in an enhancement in grain size and RMS roughness, and in the formation of a polycrystalline structure. Changes in grain size and structure have a strong impact on saturation magnetization and coercivity of the Fe_3_O_4_ thin films. Our results indicate that the combination of Ta and MgO buffer layers can influence the morphology and structure of Fe_3_O_4_ thin films and help to boost the magnetic properties.

## Experimental

RF magnetron sputtering was used at room temperature to grow magnetite films with 40 nm thickness on a variety of substrates, including SiO_2_, MgO(100), and the multilayer substrate MgO/Ta/SiO_2_/Si(100). The MgO(100) substrates were prepared by immersing them in a methanol bath at a temperature of 60 °C and drying them in N_2_ gas flow. Subsequently, the purified substrates were moved into an ultrahigh vacuum (UHV) chamber and underwent a pre-heating process at 600 °C for 30 min in order to eliminate any remaining impurities. The SiO_2_/Si(100) substrates were immersed in acetone and 2-propanol for a duration of 2 min in an ultrasonic bath. Subsequently, they were immersed in a solution of methanol at a temperature of 60 °C and then dried in N_2_ gas flow. A 5 nm thick layer of tantalum was deposited on a SiO_2_/Si(100) substrate using RF magnetron sputtering. This was followed by the formation of a 5 nm thick layer of MgO. The Fe_3_O_4_ layers were applied using RF magnetron sputtering at a base pressure of 10^−8^ Torr, employing a flow of 33 sccm of Ar gas to maintain a stable plasma. The initially deposited films were annealed at a temperature of 723 K for a duration of 2 h under a base pressure of 2.3 × 10^−8^ Torr. The Fe_3_O_4_ films were analyzed regarding their surface morphology, magnetic properties, and structural properties using atomic force microscopy (EasyScan2, Nanosurf), vibration sample magnetometry (Quantum Design magnetic property measurement system, MPMS-5XL), and X-ray diffractometry (Bruker Discover D8), respectively.

## Data Availability

The data that supports the findings of this study is available from the corresponding author upon reasonable request.
